# Leucine-Rich Repeat Kinase 2 (*Lrrk2*) Deficiency Diminishes the Development of Experimental Autoimmune Uveitis (EAU) and the Adaptive Immune Response

**DOI:** 10.1371/journal.pone.0128906

**Published:** 2015-06-11

**Authors:** Wambui S. Wandu, Cuiyan Tan, Osato Ogbeifun, Barbara P. Vistica, Guangpu Shi, Samuel J. H. Hinshaw, Chengsong Xie, Xi Chen, Dennis M. Klinman, Huaibin Cai, Igal Gery

**Affiliations:** 1 Laboratory of Immunology, National Eye Institute, National Institutes of Health, Bethesda, MD, 20892, United States of America; 2 Transgenic Section, Laboratory of Neurogenetics, National Institute on Aging, National Institutes of Health, Bethesda, MD, 20892, United States of America; 3 Cancer and Inflammation Program, National Cancer Institute, National Institutes of Health, Frederick, MD, 21702, United States of America; University of Padova, ITALY

## Abstract

**Background:**

Mutations in *LRRK2* are related to certain forms of Parkinson’s disease and, possibly, to the pathogenesis of Crohn’s disease. In both these diseases inflammatory processes participate in the pathogenic process. LRRK2 is expressed in lymphoid cells and, interestingly, *Lrrk2* (-/-) mice were reported to develop more severe experimental colitis than their wild type (WT) controls. Here, we examined the possible involvement of LRRK2 in the pathogenesis of experimental autoimmune uveitis (EAU), an animal model for human uveitis, by testing *Lrrk2* (-/-) mice for their capacity to develop this experimental eye disease and related immune responses.

**Methods:**

*Lrrk2* (-/-) mice and their WT controls (C57Bl/6) were immunized with interphotoreceptor retinoid-binding protein (IRBP) and compared for their development of EAU, delayed type hypersensitivity (DTH) by skin tests, production of cytokines in culture, and expression of interferon (IFN)-γ, interleukin (IL)-17 and FoxP3 by spleen cells, using flow cytometry. Peritoneal macrophages were examined for their production of cytokines/chemokines in culture following stimulation with LPS or the oligodeoxynucleotide CpG. The *Lrrk2* (-/-) and WT mice were also compared for their response to bovine serum albumin (BSA).

**Results:**

The *Lrrk2* (-/-) mice developed lower levels of EAU, DTH responses and cytokine production by lymphocytes than did their WT controls. Intracellular expression of IFN-γ and IL-17, by spleen cells, and secretion of cytokines/chemokines by activated peritoneal macrophages of Lrrk2 (-/-) mice trended toward diminished levels, although variabilities were noted. The expression levels of FoxP3 by Lrrk2 (-/-) spleen cells, however, were similar to those seen in WT controls. Consistent with their low response to IRBP, *Lrrk2* (-/-) mice responded to BSA less vigorously than their WT controls.

**Conclusions:**

*Lrrk2* deficiency in mice diminished the development of EAU and the related adaptive immune responses to IRBP as compared to the WT controls.

## Introduction

Leucine-rich repeat kinase 2 (LRRK2), also known as Dardarin, plays an important role in the neural system; mutations in the *LRRK2* gene are responsible for certain forms of Parkinson’s disease [1–3]. In addition, mutations in the *LRRK2* gene were found to be associated with Crohn’s disease, an inflammatory bowel disease [4–6]. The function of LRRK2 in the CNS is not known, but examination of the pathogenic process of Parkinson’s disease revealed the involvement of inflammatory processes in this condition, suggesting that a defect in the lymphoid system could play a role in the pathogenic process of this disease [7, 8]. Immune-mediated inflammation is considered to be a major pathogenic mechanism of Crohn’s disease [9, 10].

Several published studies have provided evidence to show the involvement of LRRK2 in the immune system and accumulating data on this topic are summarized in an extensive recent review by Russo et al. [7]. The *Lrrk2* gene and its protein have been detected in several cells involved in immunological and inflammatory processes, in particular B-cells, monocytes and dendritic cells [8, 11–13]. Furthermore, the expression of the LRRK2 protein and its gene were found to increase following exposure of these cells to microbial products and other pathogenic stimuli [11, 12]. Deficiency in *Lrrk2* in rats was found to perturbate the immunological homeostasis in rats [14]. he involvement of *Lrrk2* in the pathogenic process of Crohn’s disease was investigated in a study by Liu et al.[4], in which *Lrrk2* deficient mice were found to be more susceptible than their wild type (WT) controls to experimental colitis, induced by treatment with dextran sulfate sodium.

In the present study we examined the effect of *Lrrk2* deficiency on the susceptibility of mice to the induction of experimental autoimmune uveitis (EAU). EAU serves as an animal model for uveitic conditions in humans, a family of eye diseases, assumed to be immune-mediated, that includes sympathetic ophthalmia, “birdshot” chorioretinopathy, Behcet’s disease, Vogt Koyanagi Harada (VKH) disease and sarcoidosis [15, 16]. EAU is induced in mice by immunization with the retinal protein, interphotoreceptor retinoid-binding protein (IRBP). Unlike the observation with experimental colitis [1], mice deficient in *Lrrk2* were found in our study to be less susceptible than the WT controls to the induction of EAU. In addition, the *Lrrk2* (-/-) mice developed lower levels of cellular immunity against the immunizing antigen as compared to their WT controls.

## Materials and Methods

### Mice

*Lrrk2* (-/-) mice were generated as described [17] and were further backcrossed onto the C57Bl/6J background, facilitated by genome scan. The deficiency of *Lrrk2* in the deficient mice was determined by the absence of LRRK2 in tissue extracts, demonstrated by Western blotting (S1 Fig). *Lrrk2* (-/-) mice and their WT littermates, or matched WT mice from the same colony, were used at 8–16 weeks of age. All experiments were done with the approval of the Animal Care and Use Committee of the National Eye Institute, NIH.

### Induction and Evaluation of EAU

*Lrrk2* (-/-) mice and their WT controls were immunized with bovine IRBP (150 μg) and human IRBP peptide 1–20 (200 μg), emulsified with complete Freund’s adjuvant (CFA) containing 2.5 mg/ml killed *Mycobacterium tuberculosis* (DIFCO, Detroit, MI), administered subcutaneously in a volume of 0.2 ml. In addition, the mice were injected intraperitoneally with 0.2 μg pertussis toxin (List Biological Laboratories, Inc., Campbell, CA). The development of ocular inflammation was determined by histological examination on day 14 post-immunization, following euthanasia. Severity of disease, on a scale of 0–4, in half point increments, was scored as follows: focal non-granulomatous, monocytic infiltration in the choroid, ciliary body and retina were scored as 0.5. Retinal perivascular infiltration and monocytic infiltration in the vitreous were scored as 1. Granuloma formation in the uvea and retina, the presence of occluded retinal vasculitis, along with photoreceptor folds, serous detachment and loss of photoreceptors were scored as 2. In addition, retinal folding, the formation of granulomas at the level of the retinal pigmented epithelium and the development of subretinal neovascularization were scored as 3 and 4, according to the number of these pathological features.

### Immunization with Bovine Serum Albumin (BSA)

*Lrrk2* (-/-) mice and their WT controls were immunized with BSA (MP Biomedicals, Solon, OH), at 150 μg/mouse, emulsified with CFA. In addition, the mice were injected with pertussis toxin, at 0.2 μg/mouse. The immune responses against BSA of these mice were measured similarly to those for the mice immunized with IRBP, detailed below.

### Delayed Type Hypersensitivity (DTH) Skin Tests

On day 12 post-immunization mice were skin-tested by injection of the immunizing antigen, IRBP or BSA, at 10 μg or 30 μg, respectively, into their ear pinnae, in a volume of 10 μl. The ear thickness was measured before injection and 48 hrs later, using a micrometer. The response was calculated by the equation: ear thickness at 48 hrs minus thickness pre-injection.

### Production of Cytokines by Cultured Spleen Cells

Spleen cells, collected on day 14 post-immunization, were cultured in 24-well plates at 5x10^6^ cells/well in 1 ml of RPMI 1640 medium, supplemented with 2% HL-1 serum replacement (Lonza, Walkesville, MD), antibiotics, and 2-mercaptoethanol. The cultures were stimulated with bovine IRBP at 10 μg/ml, or BSA, at 5 or 50 μg/ml. Supernatants were collected following incubation for 48 hrs and their levels of interferon (IFN)-γ and interleukin (IL)-17 were determined by enzyme-linked immunosorbent assay (ELISA) kits (R&D Systems, Minneapolis, MN).

### Intracellular Expression of IFN-γand IL-17 by Spleen Cells

Spleen cells from the immunized mice collected on day 14 post-immunization were incubated for 4 hrs with PMA at 20 ng/ml and ionomycin at 1μM (both from Sigma, St. Louis, MO) in the presence of GlogiStop (BD Bioscience, San Jose, CA). According to the manufacturer’s instructions, the cells were then surface stained with antibody against CD4, conjugated with FITC and intracellular stained with APC-anti-IFN-γ and PE-anti-IL-17 (BD Bioscience, San Jose, CA). CD4^+^ cells were acquired on a MACS Quant analyzer (Miltenyi Biotec, San Diego, CA) and the data were analyzed by FlowJo software (FlowJo LLC., Ashland, OR).

### Expression of FoxP3 by Spleen Cells

Spleen lymphocyte suspensions, prepared as described above were tested for their expression of FoxP3 using flow cytometry, following the manufacturer’s protocol (eBioscience, San Diego, CA). In short, spleen cells were treated with the fixation/permeabilization buffer before intracellular staining with allophycocyanin-conjugated anti-FoxP3 antibody. Cells were acquired and data were analyzed as detailed above.

### Measurement of Cytokines and Chemokines Released by Peritoneal Macrophages

Peritoneal macrophages of *Lrrk2* (-/-) and WT controls were collected 72 hrs following induction by intraperitoneal injection of thioglycollate (Sigma) and were cultured at 2 million cells in 2 ml RPMI-1640 medium, detailed above, in 12 well plates. The macrophage cultures were stimulated with LPS (DIFCO, Detroit, MI), at 0.5 μg/ml, or with CpG oligodeoxynucleotide 1555 (5’-GCTAGACGTTAGCGT-3’), at 40 μg/ml. The supernatants were collected after 48 hrs of incubation and the levels of 14 cytokines/chemokines (CCL2/MCP-1, CXCL1/KC, G-CSF, IL-1α, IL-1β, IL-4, IL-6, IL-10, IL-12p70, IL-13, TNF-α, and CCL11/Eotaxin) were measured from the supernatants, according to the manufacturer’s specifications, using a bead-based multi-plex screening assay from R&D Systems (Minneapolis, MN) on a Luminex 100 instrument (Luminex Corp. Austin, TX). Data were analyzed using Bio-Plex Manager Pro 6.1 analysis software (Bio-Rad, Hercules, CA).

### Statistical Methods

**Fig 1:**
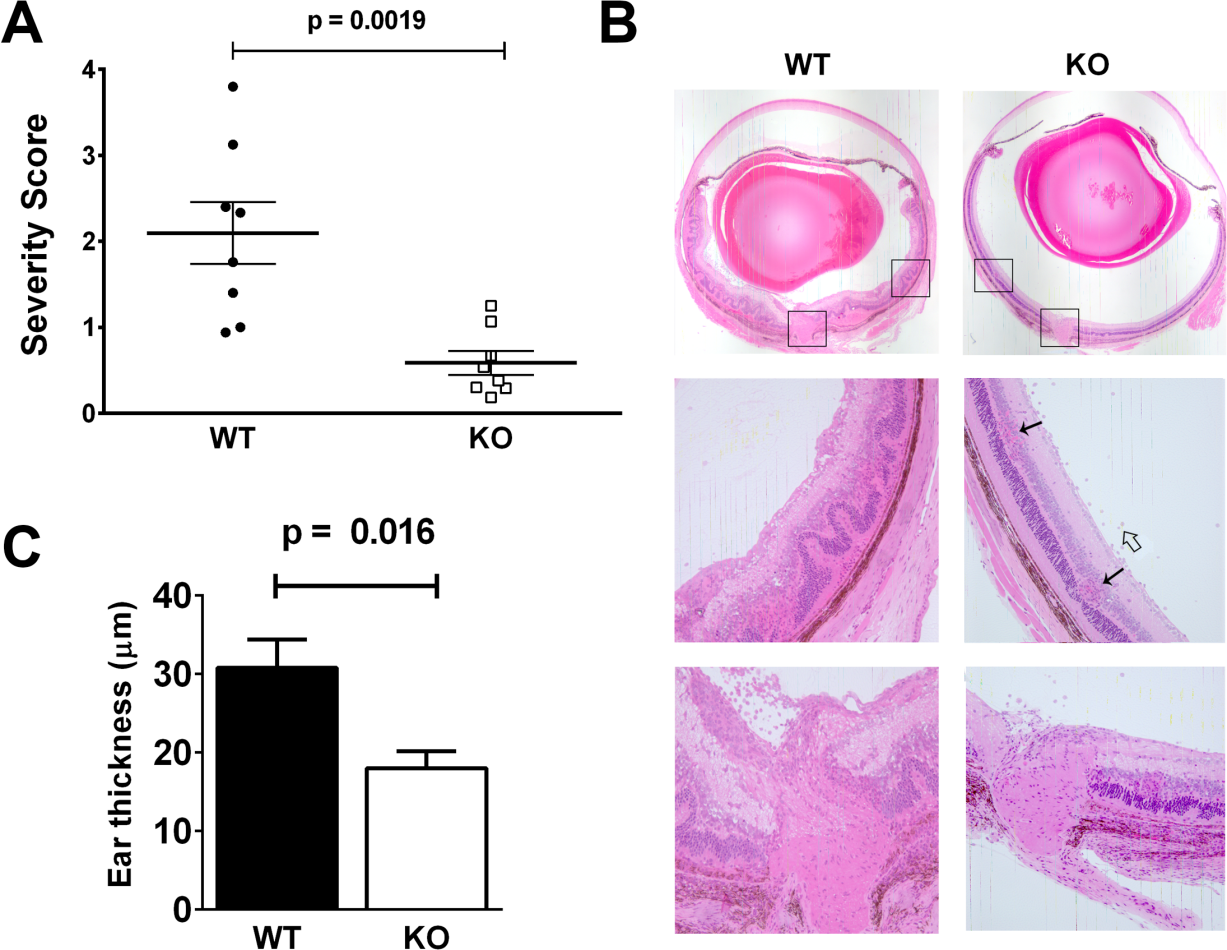
*Lrrk2* (-/-) mice develop less severe EAU and DTH responses than their WT controls. **A.** A summary of disease histological scores in eye sections of *Lrrk2* (-/-) (“KO”) and WT controls in eight repeated experiments, fourteen days post-immunization. Each dot represents the mean score of a mouse group in one experiment and the horizontal bars are the means ± SEM of *Lrrk2* (-/-) mice and their WT controls in all eight experiments. **B.** Representative histological sections of eyes of a WT and an *Lrrk2* (-/-) mice. Severe inflammation (scored at 3.5+) in the WT control eye, consisting mainly of intense infiltration of inflammatory cells in all retinal layers, as well as remarkable retinal folding. The eye of the Lrrk2 (-/-) mouse is affected by mild inflammation (scored at 1.0), that consists of inflammatory cells in the vitreous (an open arrow) and loci of infiltration into the retina (arrows). **C**. A representative DTH experiment demonstrating significantly less intense skin response in the *Lrrk2* (-/-) mouse group than in their WT controls. Similar differences between the two groups were observed in two additional experiments.

Statistical analysis was performed using GraphPad Prism 6 (GraphPad Software Inc. La Jolla, CA). Statistical significance was determined using unpaired *t* tests with p ≤ 0.05. Where non-normal distributions were compared, the Mann-Whitney test was used. The pathological scores of the WT and the *Lrrk2* (-/-) mouse groups (Fig 1A) represent the average score from each group in eight individual experiments. To allow comparison of ELISA results across multiple experiments ELISA results were normalized 0–1 with feature scaling, where:

$$X\prime =\frac{x_{i}-x_{min}}{x_{max}-x_{min}}$$

## Results

### Lrrk2 (-/-) Mice Develop Less Severe EAU than their WT Controls

We compared *Lrrk2* (-/-) mice to their WT controls for their capacity to develop EAU when immunized with IRBP. The comparison was carried out in eight repeated experiments. The disease development was determined by histological examination and the accumulated data, summarized in Fig 1A, show that the pathological ocular changes in *Lrrk2* (-/-) mice were lower than those of their WT controls. Similar to observations in other studies [18], C57Bl/6 mice vary in their susceptibility to EAU induction; yet, the difference between the accumulated EAU severity means of the deficient mice and their WT controls was significant (p = 0.0019) (Fig 1A). It is also noteworthy that in each of the eight repeated experiments, the severity means of pathological changes were lower in the deficient mice than in their corresponding WT controls. Fig 1B shows typical eye sections of an *Lrrk2* (-/-) mouse and a WT control, demonstrating severe inflammation in the control mouse eye (severity score of 3.5+) and just moderate changes in the deficient mouse eye (severity score of 1.0+).

### Lrrk2 (-/-) Mice Are Less Potent than their WT Controls in Development of DTH Response

Similar to the pathogenic process of EAU, DTH responses are mediated by Th cells and we used this immunological assay to further compare between *Lrrk2* (-/-) mice and their WT controls. The data of a representative experiment are shown in Fig 1C and demonstrate significantly stronger responses by the WT mice than by the *Lrrk2* (-/-) mice, in line with the more severe EAU changes in the WT mice.

### Proportions of Th1 and Th17 Cells in the Spleens of Immunized Lrrk2 (-/-) and WT Controls

**Fig 2:**
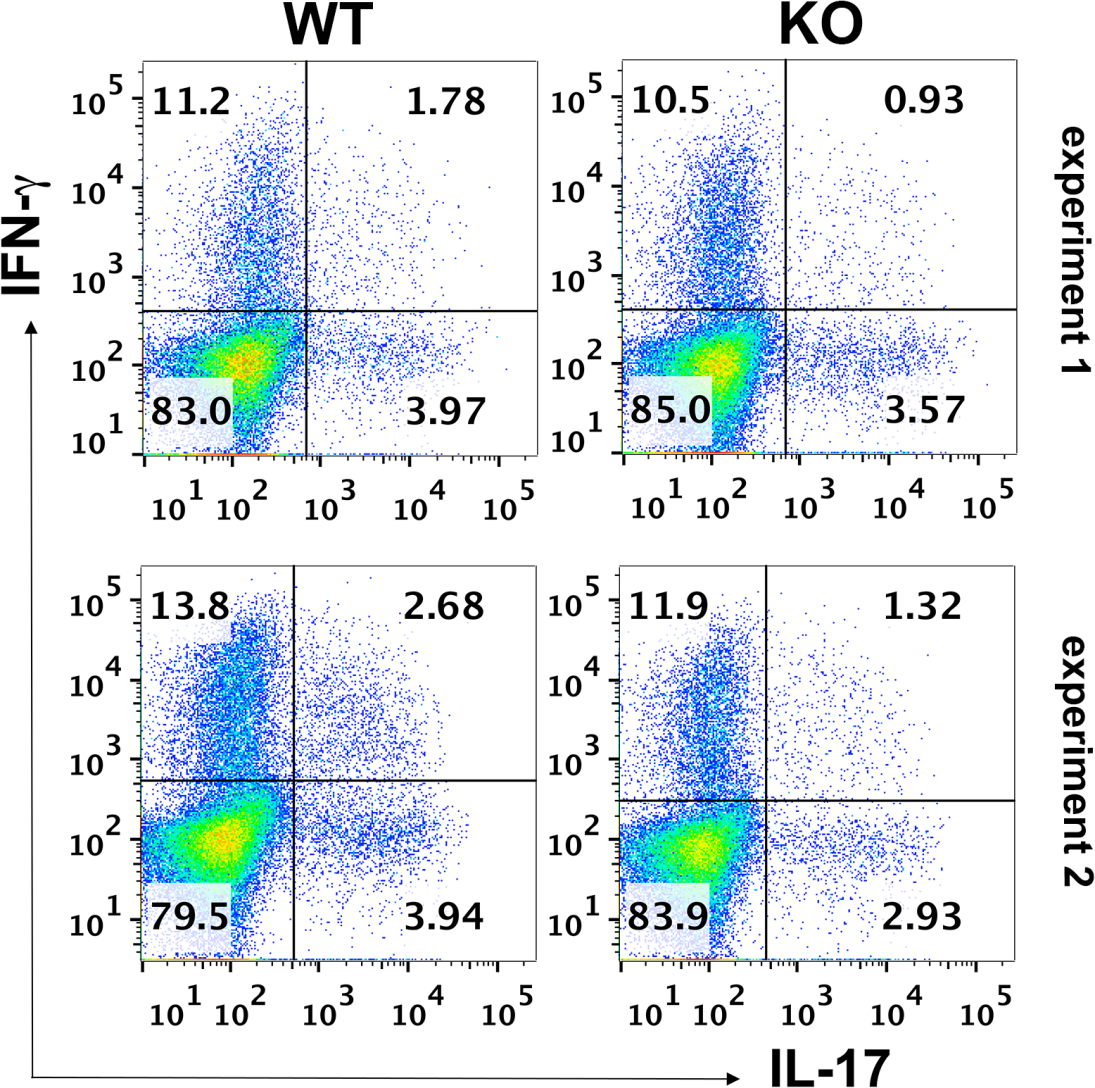
Generation of Th1 and Th17 polarized cells in *Lrrk2* (-/-) (“KO”) mice is moderately less vigorous than in their WT controls. Suspensions of pooled spleen cells of mice of the two groups, 14 days post immunization with IRBP, were examined by flow cytometry for intracellular staining with antibodies against IFN-γ or IL-17, the signature cytokines for Th1 and Th17, respectively. Data of two individual experiments.

Next, we determined the proportions of Th1 and Th17 cells in the spleens of the immunized mice, using flow cytometry to measure the proportions of cells intracellularly expressing IFN-γor IL-17, respectively. Fig 2 records data of two experiments, similarly showing slightly higher levels of cells stained for the two signature antigens in the WT mice than in the *Lrrk2* (-/-) ones.

### Lymphocytes of Lrrk2 (-/-) Mice Produce Lower Levels of IFN-γ and IL-17 than do their WT Controls

**Fig 3:**
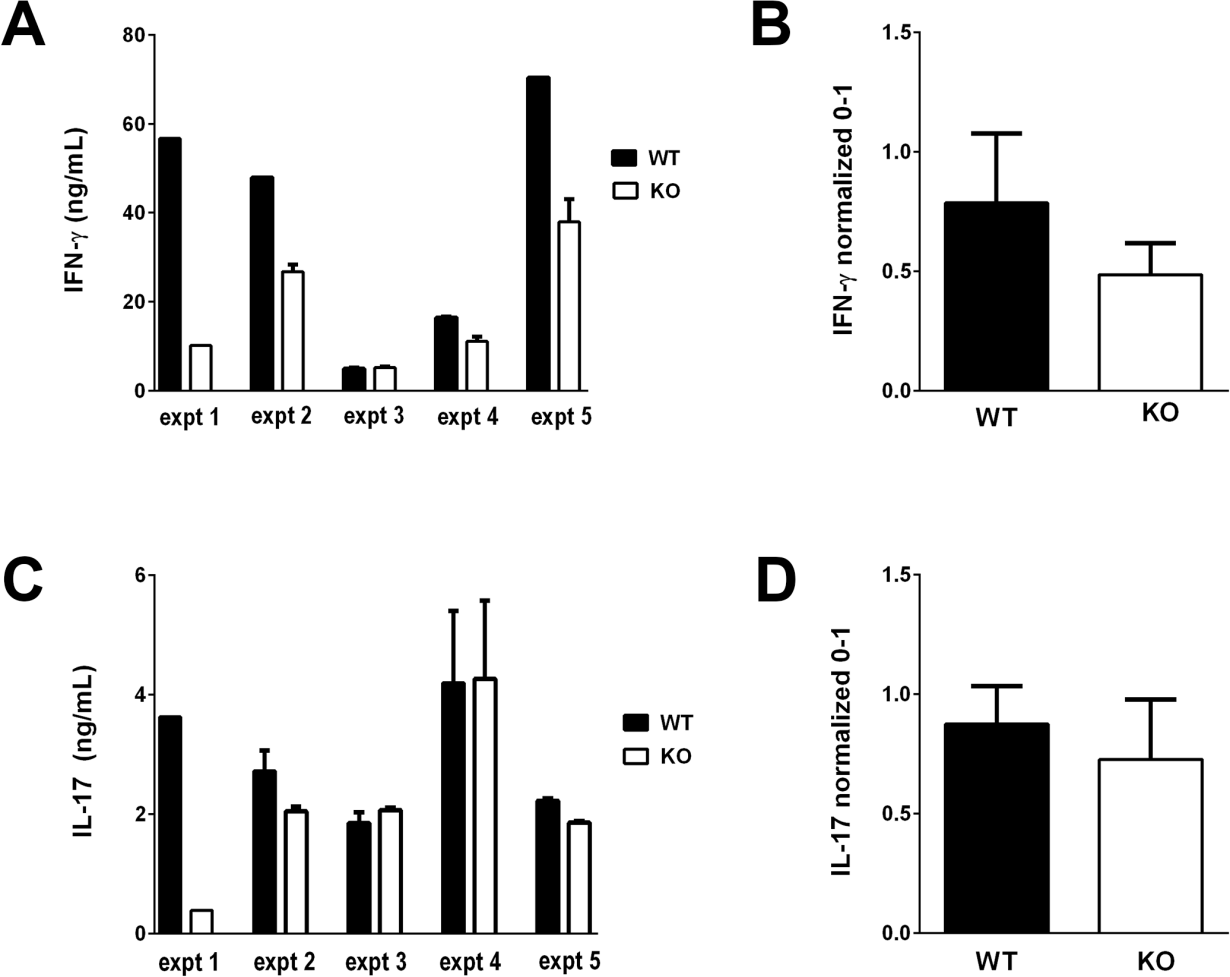
Spleen cells of *Lrrk2* (-/-) (“KO”) mice immunized with IRBP secrete lower levels of IFN-γand IL-17 than their WT controls. **A and C.** Levels of IFN-γ and IL-17 measured by ELISA in supernatants of spleen cells of five individual experiments, collected 14 days following immunization and cultured with IRBP at 10 μg/ml for 48 hrs. **B and D**. Summaries of the averaged and normalized data of the two cytokines in the five individual experiments, as detailed in the Materials and Methods section.

Pathological changes of EAU have been shown to be mediated by Th1 and Th17 cells specific against IRBP [16, 19]. To measure the specific immunological capacity of lymphocytes of the tested mouse groups, we collected their spleens and tested the splenocytes for secretion of IL-17 and IFN-γ following exposure, in vitro, to IRBP. The data of five individual experiments are recorded in Fig 3A and 3C and are summarized in Fig 3B and 3D. Higher levels of IFN-γ were measured in cultures of WT cells than in those of the Lrrk2 (-/-) in four of five experiments, whereas IL-17 production was higher in the WT cultures in only three of the five experiments. Due to variability among experiments, the differences between the *Lrrk2* (-/-) and WT controls did not reach significance.

### Proportions of FoxP3 Expressing Cells Are Similar in Spleens of Lrrk2(-/-) and WT Control Mice

**Fig 4:**
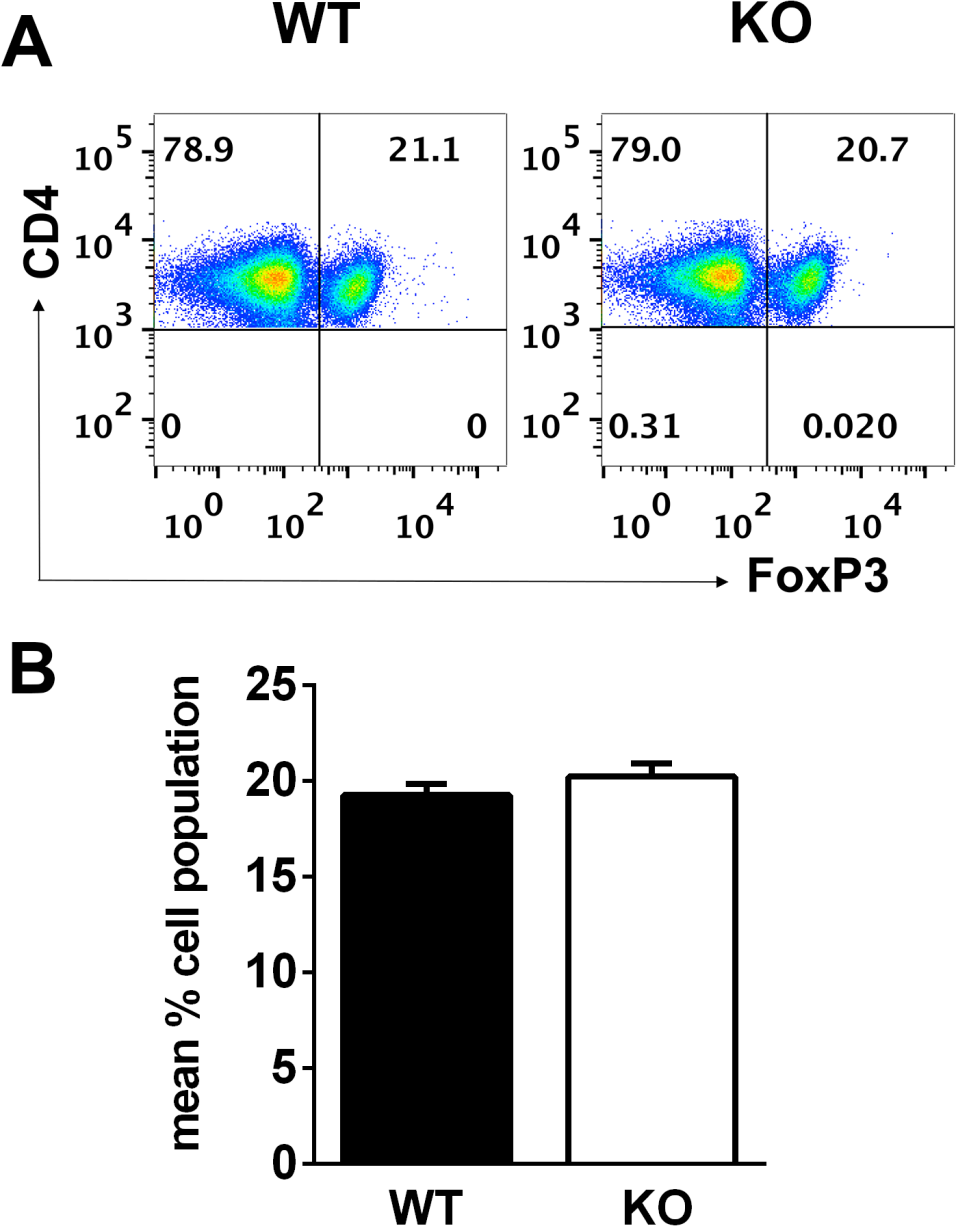
Similar levels of Treg cells expressing FoxP3 in spleens of *Lrrk2* (-/-) (“KO”) and their WT controls. Spleens from mice of the two groups were collected 14 days post immunization, pooled, and their cells were examined by flow cytometry for expression of FoxP3. **A**. A representative experiment. **B**. Mean +/- SEM of proportions of FoxP3 cells in spleens of mice of the two groups from five individual experiments.

To examine the possibility that the inferior immune responsiveness in *Lrrk2* (-/-) mice is due to increased levels of Treg cells, we compared by flow cytometry the proportions of CD4 cells expressing FoxP3 in spleens of the two mouse groups. FoxP3 is a transcription factor specifically expressed by Treg cells [20, 21]. Fig 4A shows the flow cytometric data of a representative experiment and the data of this and four other experiments are summarized in Fig 4B. Just minute differences were noted between the *Lrrk2* (-/-) and their WT controls in the proportions of FoxP3 expressing cells in their spleen, indicating that the lower immune response in the *Lrrk2* (-/-) mice is not due to increase in proportions of Treg cells.

### Comparison between Peritoneal Macrophages of Lrrk2 (-/-) and WT Control Mice for their Response to TLR Ligands in Culture

**Fig 5:**
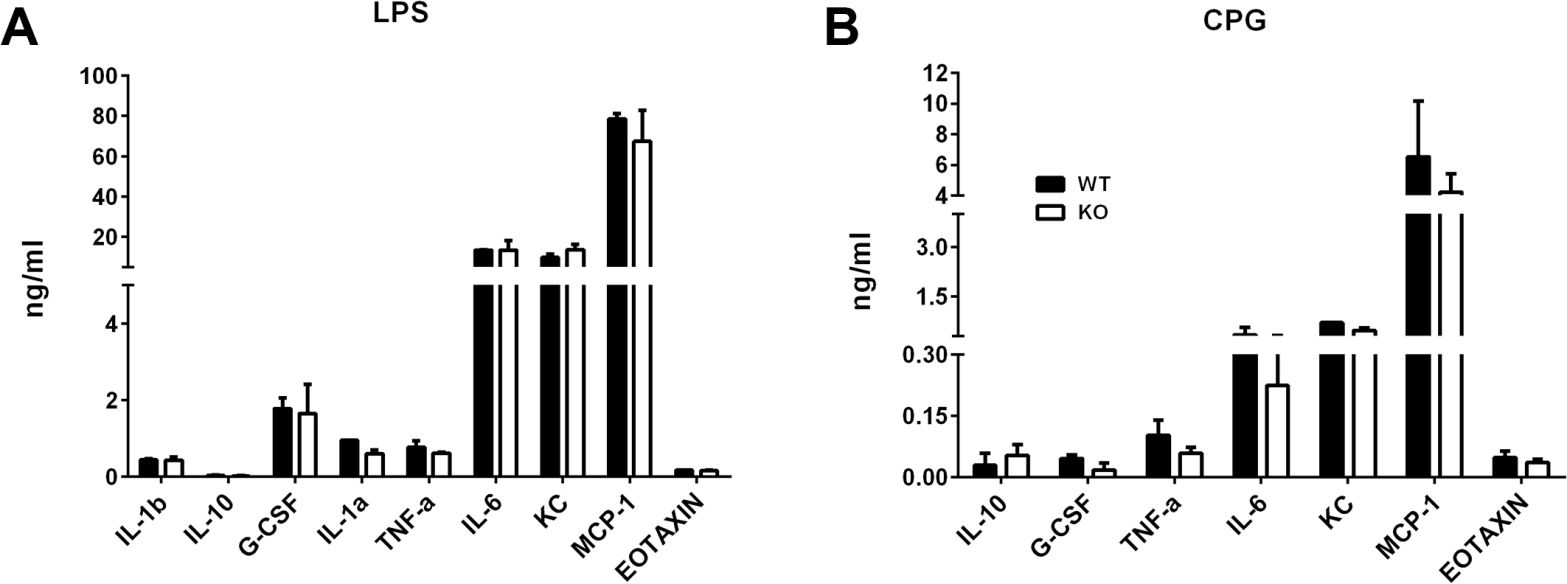
Minute differences seen in the profiles of cytokines/chemokines released by peritoneal macrophages of the *Lrrk2* (-/-) (“KO”) and their WT controls, cultured with LPS (0.5 μg/ml) or CpG (40 μg/ml) for 48 hrs. The levels of the released products were measured by a bead-based multi-plex screening assay. The assay measured secretion of 14 analytes, as detailed in the Materials and Methods section, but molecules with undetectable levels were not included in the figure. The figures show combined data collected in two individual experiments and combined for presentation.

In addition to their participation in phagocytosis, macrophages play major roles in the immune system as producers of cytokines and chemokines. To compare between macrophages of *Lrrk2* deficient mice and WT controls, we collected their induced peritoneal macrophages and measured the levels of certain cytokines and chemokines released in cultures stimulated with two TLR ligands, LPS and CpG [22]. The data collected in two separate experiments are combined in Fig 5 and show only small differences in levels of the majority of tested molecules produced by macrophages of the two mouse groups when stimulated by LPS or CpG. These observations are in line with findings of a study by Dzamko, et al [23]. It is of interest, however, that the mean levels of TNF-α were higher in supernatants of WT than of *Lrrk2* (-/-) cells when cultured with either LPS or CpG.

### The Immune Responsiveness of Lrrk2 (-/-) Mice to an Antigen other than IRBP, Namely BSA, is also Lower than that of the WT Controls

**Fig 6:**
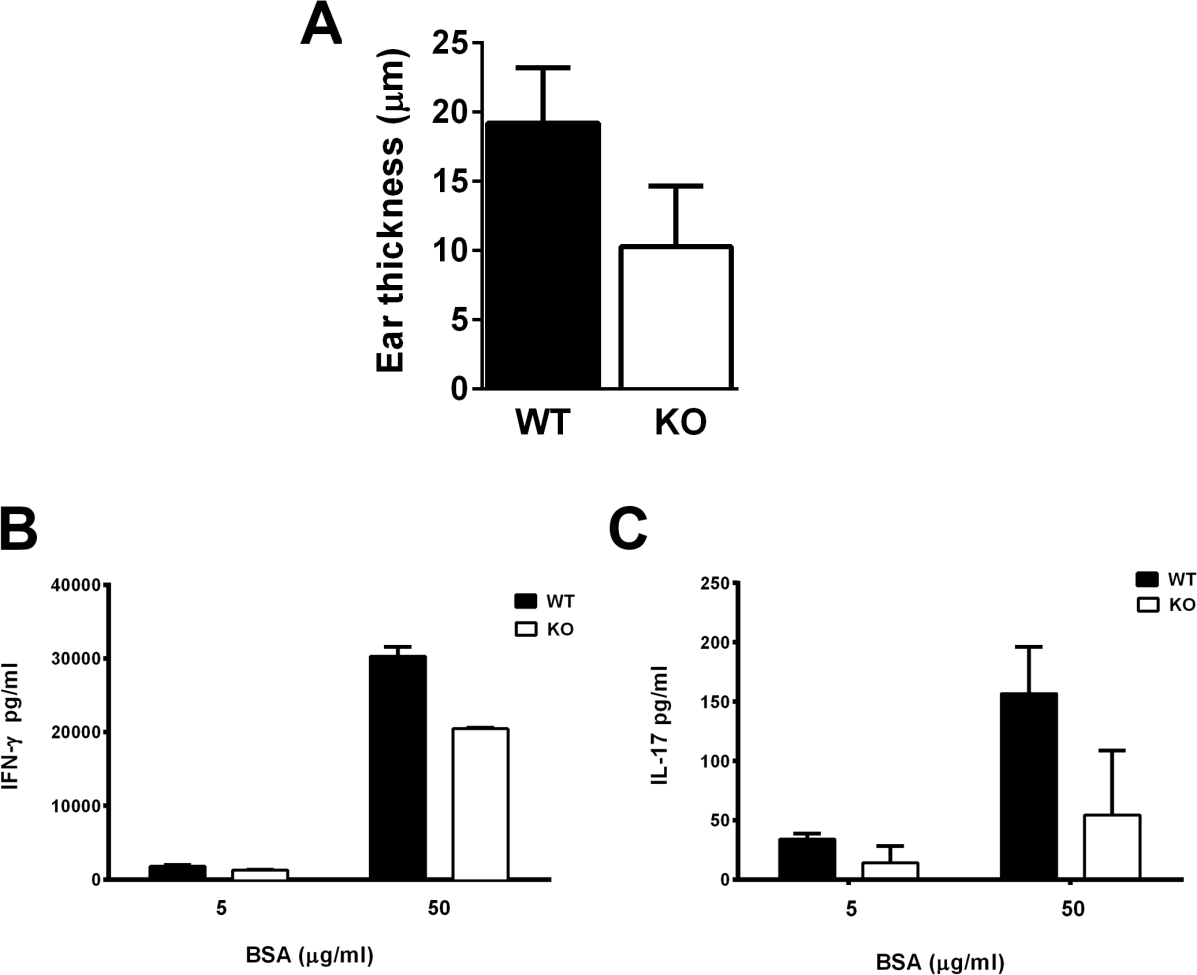
Immune responses to BSA by *Lrrk2* (-/-) mice are lower than those of their WT controls. Mice of the two lines were immunized with BSA and tested for DTH skin response with BSA (**A**) and secretion of IFN-γ (**B**) and of IL-17 (**C**) by spleen cells incubated with BSA. Data of a representative experiment; similar trends were found in a repeated experiment.

To examine the specificity of the reduced immune response of *Lrrk2* (-/-) mice we compared groups of *Lrrk2* (-/-) and WT controls for their response to BSA. The immunized mice were tested for their DTH response and spleen cells were examined for their production of IFN- γ and IL-17 following exposure to BSA in culture. Data collected with these assays are recorded in Fig 6A and 6B and 6C, respectively, and show that, similar to their reduced response to IRBP, *Lrrk2* (-/-) mice reacted to BSA with levels lower than those of their WT controls.

## Discussion

Data recorded here show that the capacity of *Lrrk2* (-/-) mice to develop EAU following immunization with IRBP is lower than that of their WT controls. This observation is in contrast to that of Liu et al [1], who found that *Lrrk2* (-/-) mice developed more severe experimental colitis than their WT controls. The *Lrrk2* (-/-) mice in the two studies were of the same colony, and the remarkable difference in their susceptibility to the two diseases could be mainly attributed to differences in the pathogenic mechanisms that mediate these two experimental diseases. EAU is initiated by the adaptive immune system [16, 24], whereas innate immunity plays a role in the pathogenesis of experimental colitis [4, 25, 26].

In order to learn about possible mechanistic immunological differences between the *Lrrk2* (-/-) mice and their WT controls, we compared the two groups of animals by a battery of immunological assays related to the pathogenic process of EAU. A correlation was observed between the susceptibility of mice to EAU induction and their DTH skin test response, another process mediated by Th cells. Both Th1 and Th17 are involved in the pathogenesis of EAU [16, 19, 27] and we examined these two populations in the *Lrrk2* deficient mice and their WT controls by two assays, i.e., their proportions among the spleen lymphocytes in the immunized mice and their capacity to produce in culture their signature cytokines, IFN-γ and IL-17, respectively. The proportions of cells producing IFN-γ or IL-17, determined by flow cytometry, were found to be slightly higher in spleens of WT mice than in the *Lrrk2* (-/-) mice. Production of cytokines by cultured spleen cells, measured by ELISA, revealed that lymphocytes from the *Lrrk2* (-/-) mice were quite consistent in producing lower levels of IFN- γ as compared to their WT controls. The difference between the two groups of mice was less consistent for the release of IL-17, a finding that could be attributed to the production of this cytokine by non-Th17 cells, in particular γδT-cells and innate lymphoid cells [28]. It is conceivable that these non-Th17 cells are less affected by the *Lrrk2* deficiency than Th17 cells.

Another family of cells involved in the immune response tested in this study were peritoneal macrophages. When stimulated in culture with LPS or CpG, macrophages from *Lrrk2* (-/-) mice resembled those from the WT controls in their levels of production of the majority of tested cytokines and chemokines (Fig 5). It is of note that Liu et al. [4] found that bone marrow-derived macrophages from Lrrk2 (-/-) produced higher levels of several cytokines than did the WT control macrophages, when stimulated with Zymosan. The difference between the two studies could be due to different populations of macrophages used in the two studies and different stimuli; as shown by Liu et al, the patterns of cytokine release depends on the stimulus [4]. Interestingly, LRRK2 inhibition or deficiency was reported to attenuate the pro-inflammatory activities of microglia, the macrophage-like cells of the CNS, including their cytokine production [29, 30].

Reduced immune response may be caused by an increase in proportion of Treg cells [20, 21]. We tested this hypothetical situation by comparing the number of cells that express FoxP3 in the spleens of *Lrrk2* (-/-) mice and WT controls. Essentially no differences were detected between the two mouse groups by this assay (Fig 4), indicating that the reduced immune responsiveness by *Lrrk2* (-/-) mice cannot be attributed to increased activity of Treg cells.

Our finding that the *Lrrk2* (-/-) mice also respond to BSA by levels lower than those of their WT controls further indicates that deficiency in *Lrrk2* affects the adaptive immune response of the deficient mice. It is obviously of interest that the *Lrrk2* (-/-) mice showed increased susceptibility to experimental colitis [4] and enhanced levels of innate immune response. Additional studies are needed to further investigate the impact of *Lrrk2* deficiency on the immune response, particularly in view of the *LRRK2* gene’s involvement in the pathogenesis of Parkinson’s disease, a condition in which inflammation plays an important role.

## Supporting Information

S1 Fig Lrrk2 (-/-) mice are deficient in Lrrk2 protein. Brain striatum tissues collected from 1 month old Lrrk2 (-/-) (“KO”) mice and their wild type controls (“nTg”) were used to obtain cytoplasm-enriched fragments (“Cyto”) and nuclei-enriched fragments (“Nu”). Western blotting was performed with anti-LRRK2 (Abcam, ab133475 [MJFF3 (c69-6)]) and anti-GAPDH (Sigma, G8795).
(TIF)
