# Supplementary figures and images for: Leucine-Rich Repeat Kinase 2 (Lrrk2) Deficiency Diminishes the Development of Experimental Autoimmune Uveitis (EAU) and the Adaptive Immune Response
Source: PLoS One. 2015 Jun 11;10(6):e0128906. doi: 10.1371/journal.pone.0128906 (PMC4465928; doi:10.1371/journal.pone.0128906)

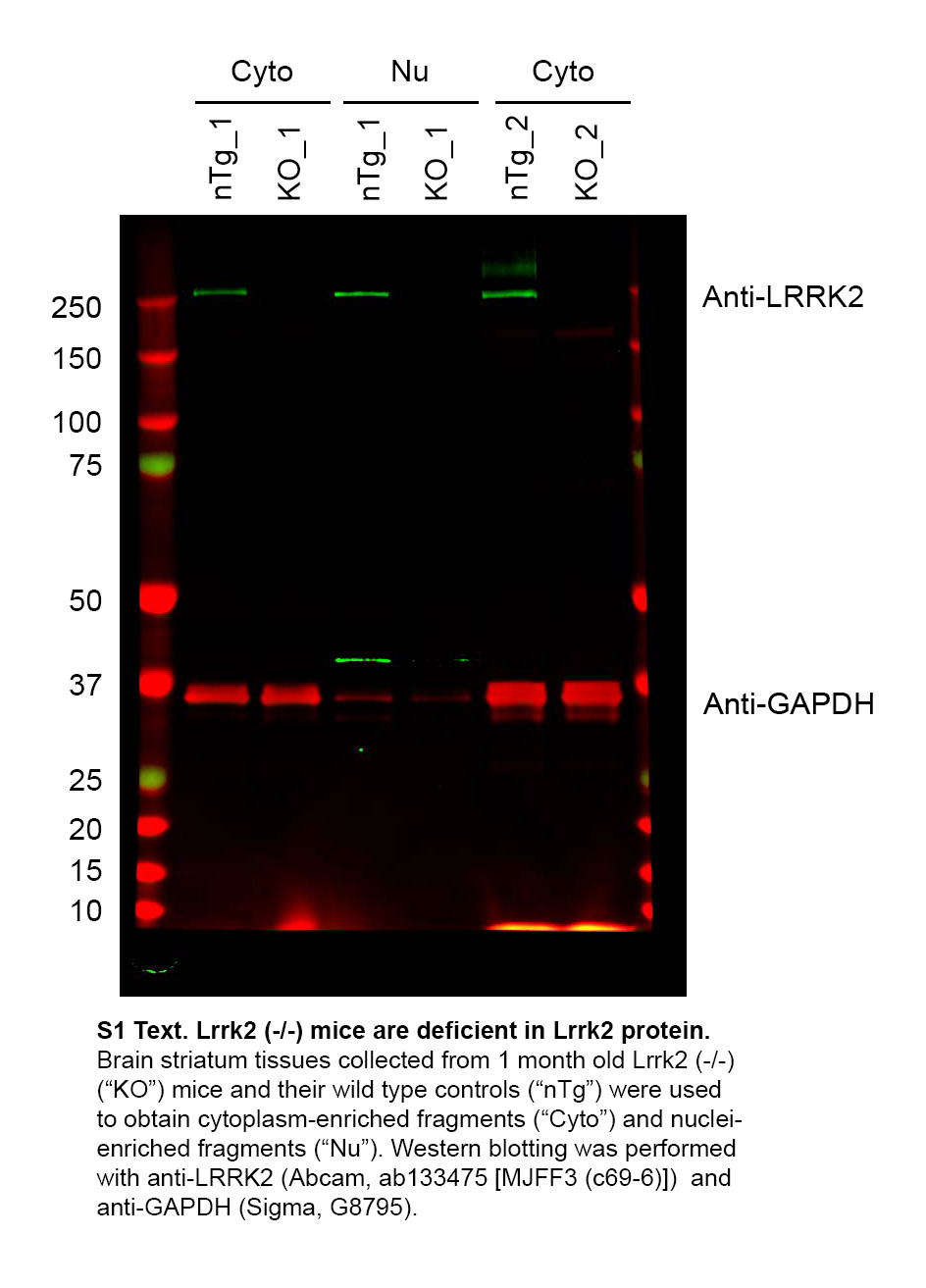

Supplement: S1 Fig — Brain striatum tissues collected from 1 month old Lrrk2 (-/-) (“KO”) mice and their wild type controls (“nTg”) were used to obtain cytoplasm-enriched fragments (“Cyto”) and nuclei-enriched fragments (“Nu”). Western blotting was performed with anti-LRRK2 (Abcam, ab133475 [MJFF3 (c69-6)]) and anti-GAPDH (Sigma, G8795). (TIF) [file pone.0128906.s001.tif]
